# Genome-Wide Analysis of TCP Transcription Factors and Their Expression Pattern Analysis of Rose Plants (*Rosa chinensis*)

**DOI:** 10.3390/cimb45080401

**Published:** 2023-07-31

**Authors:** Qingcheng Zou, Qing Dong, Danqing Tian, Lihui Mao, Xuerui Cao, Kaiyuan Zhu

**Affiliations:** Zhejiang Institute of Landscape Plants and Flowers, Hangzhou 311251, China; zouqc@zaas.ac.cn (Q.Z.); dongq@zaas.ac.cn (Q.D.); tdqing@zaas.ac.cn (D.T.); mlh@mail.zaas.ac.cn (L.M.)

**Keywords:** TCP gene family, phylogenetic analysis, synteny, gene expression, rose

## Abstract

The plant-specific transcription factor TEOSINTE BRANCHED, CYCLOIDEA, AND PROLIFERATING CELL FACTOR (TCP) gene family plays vital roles in various biological processes, including growth and development, hormone signaling, and stress responses. However, there is a limited amount of information regarding the TCP gene family in roses (*Rosa* sp.). In this study, we identified 18 TCP genes in the rose genome, which were further classified into two subgroups (Group A and Group B) via phylogenetic analysis. Comprehensive characterization of these TCP genes was performed, including gene structure, motif composition, chromosomal location, and expression profiles. Synteny analysis revealed that a few TCP genes are involved in segmental duplication events, indicating that these genes played an important role in the expansion of the TCP gene family in roses. This suggests that segmental duplication events have caused the evolution of the TCP gene family and may have generated new functions. Our study provides an insight into the evolutionary and functional characteristics of the TCP gene family in roses and lays a foundation for the future exploration of the regulatory mechanisms of TCP genes in plant growth and development.

## 1. Introduction

Transcription factors (TFs) bind to specific sequences in the upstream promoter of the target gene through their specific binding domain, influence target gene expression, and play an important role in plant growth and development. Transcription factors respond to stimuli or inhibitory signals [1]. The TEOSINTE BRANCHED, CYCLOIDEA, AND PROLIFERATING CELL FACTOR (TCP) transcription factor family is one of the major TF families in plants [2]. TCP is named after four proteins that perform different roles in plant growth and development: cycloidea (*CYC*) from Antirrhinum majus [3,4]; Teosinte branched 1 (*TB1*) in Zea mays, a cycloidea homologous gene [5,6]; and proliferating cell factors 1 and 2 (*PCF1*/*2*) from rice, which bind to proliferating cell nuclear antigen (PCNA) [7]. These four genes possess a common TCP domain and 59 amino acids, which form a basic-helix-loop-helix (bHLH) structure [8].

The TCP domain is important for DNA binding, protein–protein interaction, and subcellular localization [9,10]. According to the conserved domain, the TCP gene family can be divided into two classes: class I and class II. Class I, also called the PCF class or TCP-P class, always binds to the GGNCCCAC sequence. Class II, also named the TCP-C class, always binds to the GTGGNCCC sequence and is further subdivided into CIN and CYC/TB1 subclasses [11]. It has been well documented that the TCP gene family regulates multiple biological processes [12], including seed germination [13], branching [14], leaf morphogenesis [15], flower development [16], plant cell wall formation [15], mitochondrial biogenesis [17], gametophyte development [18], hormone pathway processes [19], flavonoid synthesis [20], circadian clock processes [21], and the adversity stress responses [19,22]. Class I TCP members often play an important role in cell growth and proliferation [23,24] as well as fruit development and ripening [25] during plant development [26]. By contrast, CIN-like subclade genes also play an important role in lateral organ development, while the CYC/TB1 subclade genes often control axillary meristem development. In *Arabidopsis*, five CIN-like *TCP* members (*AtTCP2*, *AtTCP3*, *AtTCP4*, *AtTCP10*, and *AtTCP24*) were reported to play a role in leaf morphogenesis [27]. The CYC/TB1 subclade members, *Arabidopsis AtTCP18* and *AtTCP12* and tomato *SlTCP7*, regulate the branch number by affecting gibberellin signals [14,28].

Recently, based on the development and application of whole-genome sequencing technologies, a number of TCP proteins have been identified in multiple plants, such as Arabidopsis [29], rice [29], tomatoes [25], apples [30], cotton [31], potatoes [32], water melons [33], grapes [7], tea [34], spring orchids [35], and rye [1]. However, the *TCP* gene family has not been systematically identified in *Rosa chinensis*.

*Rosa chinensis* is a well-known traditional horticultural crop that is grown around the world due to its high economic and ornamental value [36]. Using diploid *Rosa chinensis* ‘Old Blush’ genome sequencing, transcription factors identification is possible [37].

In our present study, we identified eighteen TCP genes in *Rosa chinensis* ‘Old Blush’ and employed systematic bioinformatics analysis to study TCP gene family members using genome sequencing. For TCP member identification, we analyzed the gene structure, motif composition, chromosomal location, and synteny of rose. In addition, the TCP genes expression in different tissues was analyzed based on transcriptome data, and eight TCP genes were selected for expression analysis via a quantitative real-time polymerase chain reaction (qPCR). This study provides details about the evolutionary and functional characteristics of the TCP gene family in *Rosa chinensis* and lays a foundation for the future functional characterization of TCP genes.

## 2. Methods

### 2.1. Sequence Identification and Annotation of TCP Genes in Rosa chinensis

The TCP domain (PF03634) of the hidden Markov model (HMM) (version 3.1b2, http://hmmer.org/, accessed on 14 March 2023) profile was obtained from the protein family database (Pfam) (http://pfam.sanger.ac.uk/, accessed on 14 March 2023) website. The *Rosa chinensis* genome, GFF3 file, CDS sequences, and protein sequences were downloaded from the ensemble plant website (http://plants.ensembl.org/index.html, accessed on 14 March 2023). Similarly, genome, GFF3 file, CDS sequences, and protein sequence data for *Malus domestica Golden*, *Prunus mume*, *Prunus persica*, *Prunus dulcis*, and *Prunus avium* were also downloaded from the ensemble plant website. TCP genes were searched in the database using HMMER (version 3.1b2, http://hmmer.org/, accessed on 14 March 2023), and the e-value was set to 1 × 10^−5^. Clustalw (version 2.1) was used for multisequence alignment to build a new hidden Markov model file, which was used as a query. A cutoff value (0.01) was used to filter sequences from the new hidden Markov model file and delete duplicates in these sequences. After the identification of TCP genes using HMMER, all of the candidate genes were examined using the Conserved Domain Database (CDD) (https://www.ncbi.nlm.nih.gov/Structure/cdd/wrpsb.cgi, accessed on 1 April 2023) to verify the core sequences of TCPs. Finally, the TCP genes were identified after the comprehensive curation of the rose genome. All the TCP genes from six species (*Malus domestica Golden*, *Prunus mume*, *Prunus persica*, *Prunus dulcis*, *Prunus avium,* and *Arabidopsis thaliana*) are named in Supplementary Table S1.

### 2.2. Physicochemical Analysis and Prediction of TCP Gene Family Members

The chromosome location, strand, length of coding sequences (CDS), and the number of amino acids of the rose TCP genes were obtained from gff3 file which was downloaded EnsemblPlants (http://plants.ensembl.org/index.html, accessed on 4 April 2023). In addition, Mw and theoretical pI values for rose TCPs were computed using the ProtParam tool (https://web.expasy.org/protparam/, accessed on 5 April 2023), and the predicted subcellular location information was retrieved with Plant-mPLoc (http://www.csbio.sjtu.edu.cn/bioinf/plant-multi/, accessed on 5 April 2023).

### 2.3. Phylogenetic Analysis of the TCP Genes

The amino acid sequences of TCP proteins from *Malus domestica Golden*, *Prunus mume*, *Prunus persica*, *Prunus dulcis*, *Prunus avium*, *Arabidopsis thaliana* and *Rosa chinensis* were aligned using MAFFT (version 7.0). The parameters of the MAFFT software were the Gap opening penalty, 1.53; offset value, 0.0; and scoring matrix for amino acid sequences, BLOSUM62. A phylogenetic tree was constructed using IQ-TREE software (multicore version 1.6.12). The best-fit model of the trees was JTT + F + R5 and VT + F + R3 for six species and roses only [38]. Similar to the Shimodaira–Hasegawa method, a phylogenetic assessment was conducted using ultrafast bootstraps, and 1000 replicates each were used. The tree file was visualized using FigTree (version 1.4.3, http://tree.bio.ed.ac.uk/software/figtree/, accessed on 8 April 2023).

### 2.4. Chromosomal Distribution and Gene Structure Analysis for TCP Genes

The Gff3 file was used to analyze the transcript structure. The Gtf file was extracted using the gffread command from the cufflinks package (version 2.2.1). Then, the position information for TCP gene family members was obtained from the gtf file. Samtools (Version 1.7) was used to extract the chromosome length from the *Rosa chinensis* genome. The MapGene2Chrom website (http://mg2c.iask.in/mg2c_v2.1/, accessed on 31 March 2023) was used to visualize chromosomal distribution analysis results [39]. The Gtf file was also used to obtain TCP gene structure information. Finally, MEME (version 5.1.0) [40] was used for gene motif location analyses. The parameter set was a motif length from 6 to 50 residues, with a maximum of 10 motifs and a maximum size of 60,000. The visualization of exon-intron distribution and motif location of the TCP genes in rose was performed using the Gene Structure Display Server (GSDS: http://gsds.cbi.pku.edu.cn, accessed on 31 March 2023) online tool. 

### 2.5. Synteny Analysis of TCP Genes

For collinearity analysis, we used the Multiple Collinearity Scan toolkit (MCScanX: https://github.com/tanghaibao/jcvi/wiki/MCscan-(Python-version), accessed on 10 April 2023) to analyze the gene duplication events [41].

### 2.6. Gene Expression Patterns Based on Transcriptome Data

Raw transcriptome data from nine different tissues (prickles, stamens, leaves, stems, roots, and ovaries) were downloaded from NCBI under BioProject PRJNA546486. SRA files were converted to FASTQ files using the SRA toolkit (http://www.ncbi.nlm.nih.gov/Traces/sra/, accessed on 24 February 2022). Then, FastQC software (version 1.12.1) was used to assess the read quality, and Trimmomatic was used to discard low quality portions of reads (QUALITY: 15; LEADING: 20; TRAILING: 20; MINLEN: 50; SLIDINGWINDOW: 5: 20). The reads were subsequently mapped to *R. chinensis* ‘Old Blush’ genome with HISTA2 (version 2.1.0), and bam files were processed to quantitatively analyze the gene expression using StringTie (version v2.2.0) with default parameters. Fragments per kilobase per million mapped reads (FPKM) were used to evaluate the relative transcription of eighteen TCP members. A heat map of the expression profiles of TCP genes in different tissues was drawn using the online software heatmapper (http://www.heatmapper.ca/expression/, accessed on 4 May 2023), using the average linkage clustering method and Pearson distance measurement method. 

### 2.7. qPCR for Certification

Roots, stems, leaves, ovaries, prickles, and stamens were sampled for TCP genes expression analysis via qPCR. All samples were immediately frozen in liquid nitrogen for two hours and stored at −80 °C before use. Total RNA was extracted using TRIPure reagent (Aidlab Biotechnologies, Beijing, China). First-strand cDNA was synthesized using the ReverTra Ace qPCR RT Master Mix (TOYOBO, Shanghai, China). The Step One Plus^TM^ Real-Time PCR Instrument Thermal Cycling Block (Thermo Fisher Scientific Biotechnology, Shanghai, China) was used for qPCR analysis. Eight genes were selected to study the sequencing data. The primes are listed in Supplementary Table S2. The thermos cycle was set as follows: 95 °C for 30 s; 40 cycles of 95 °C for 10 s; 60 °C for 30 s. *RcUbi* was used as an internal control. The 2^−∆∆CT^ method was employed to analyze relative gene expression levels using the mean values of three replicates.

## 3. Results

### 3.1. Identification of TCP Proteins in Roses

Eighteen members of the TCP gene family were identified and given a name in the rose genome. For the analysis of TCP gene family members, we extracted the TCPs protein sequences and predicted the molecular weight (MW), theoretical pI (pI), and subcellular localization (Table 1) of the TCP proteins. For eighteen TCP proteins, the largest protein was PRQ40431, with 448 amino acid residues, and the smallest protein was PRQ29084, with 246 amino acid residues. The TCP proteins’ molecular weights range between 28,087.84 and 50,039.06 Da. Many TCPs have been confirmed to be nuclear proteins, such as AtTCP5 and AtTCP17 [42]; therefore, the subcellular localization results show that TCP proteins are all nuclear proteins, indicating that TCP proteins are likely to function in the nucleus. Due to their differences in length, PI, and molecular weight, it is suggested that there may be different groups of TFs for TCP proteins.

### 3.2. Chromosomal Distribution of Rose TCP Genes

**Figure 1:**
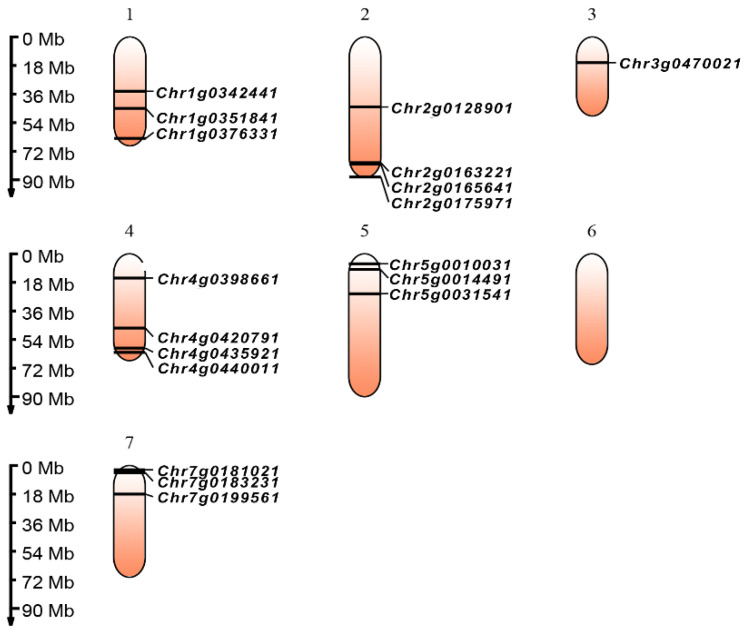
The chromosomal distribution of TCP genes in roses. TCP genes distribution in the six chromosomes. Numbers at the left stand for the length of the chromosomes and also provide the locations of these genes on their chromosomes.

Based on the whole-genome data for the roses, the precise chromosome physical locations of all TCP genes were determined, as shown in Figure 1. The rose genome is composed of seven chromosomes; the lengths of all the rose chromosomes range from 49.7 MB to 90.0 MB. In the chromosome location results of the rose genome, eighteen genes are distributed on six chromosomes, with only chromosome 6 lacking TCP genes. There are four genes on chromosomes 2 and 4, three genes on chromosomes 1, 5, and 7, and only one gene on chromosome 3.

### 3.3. Phylogenetic Analysis of TCP Genes

**Figure 2:**
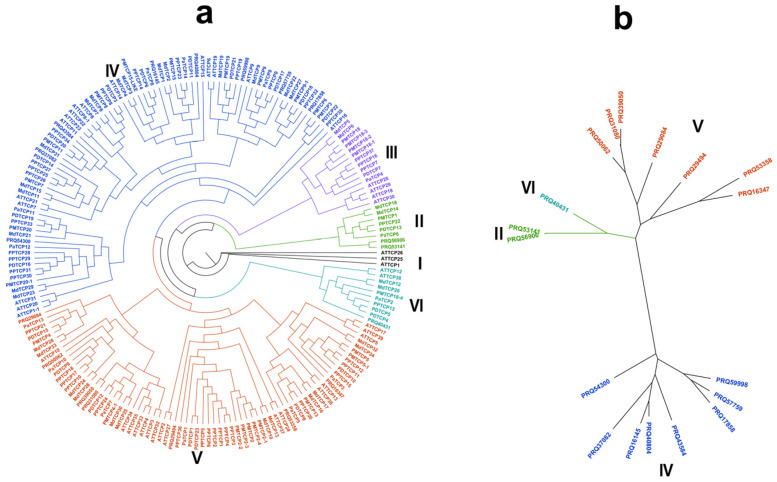
Phylogenetic tree of TCP proteins. (**a**) Phylogenetic tree of roses, containing *Malus domestica Golden*, *Prunus mume*, *Arabidopsis thaliana*, *Prunus persica*, *Prunus dulcis* and *Prunus avium* TCP members. Different-colored arcs represent different groups of TCP proteins. TCP proteins from *Malus domestica Golden*, *Prunus mume*, *Arabidopsis thaliana*, *Prunus persica*, *Prunus dulcis*, and *Prunus avium* with the prefixes “Md”, “Pm”, “AT”, “Pp”, “Pd”, and “Pa”, respectively; (**b**) TCP proteins phylogenetic tree of roses.

To investigate the TCP genes’ phylogenetic relationship for roses, we applied TCP protein sequences of *Malus domestica Golden*, *Prunus mume*, *Arabidopsis thaliana*, *Prunus persica*, *Prunus dulcis*, and *Prunus avium* to construct the phylogenetic tree. As shown in Figure 2a, 194 TCP proteins were classified into two subclasses (Group A and Group B). We divided Group A into class I–class III and Group B into class IV–class VI. There are three rose TCP proteins in Group A, including two in subclass I and one in subclass III. In Group B, there are seven rose proteins in subclass IV and eight in subclass V. In addition, subclass II in Group A and subclass VI in Group B contain no rose TCP proteins (Figure 2b).

### 3.4. Intron-Exon Distribution and Motif Analyses of TCP Genes

**Figure 3:**
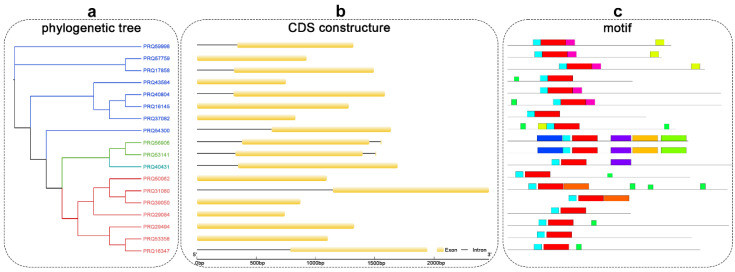
Phylogenetic tree, motif locations and gene structures of TCP proteins in roses. (**a**) The phylogenetic tree of TCP proteins in roses. The TCPs were divided into four groups. (**b**) Gene structure of TCPs. Black lines indicate introns; yellow boxes indicate exons; the genomic length is indicated at the bottom. (**c**) Motif composition of TCP proteins. Different colored boxes indicate motifs 1–10.

The CDS structure of the eighteen TCP members, including their intron-exon distribution, was studied to further verify the evolutionary and phylogenetic relationships between members of the TCP family. The CDS structure of the TCP family is relatively simple, with 38.9% of the CDSs containing one intron and one exon, 50% of the CDSs having only one exon, and 11.1% of the CDSs having two exons and two introns (Figure 3b).

**Figure 4:**
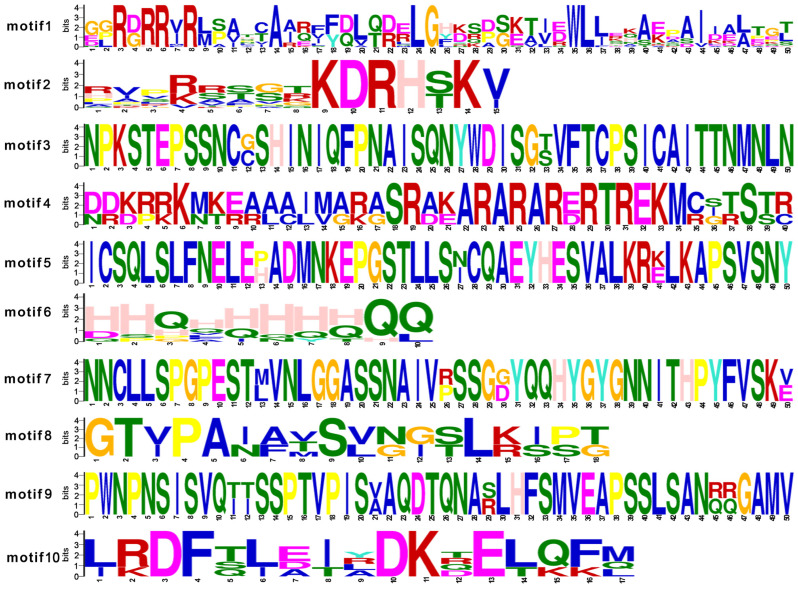
Information for each motif. Different colors distinguish amino acid residues, and the size of the letter is proportional to the frequency of occurrence of amino acid residues at the location. The Unit is bits in the figure.

In addition, we used MEME to detect the conserved motifs in eighteen TCP proteins from roses (Figure 3c). Motif 1 and motif 2 correspond to the HXXXD and DFGWG domains, respectively. Motif 6 was found in only 38.9% of the rose proteins belonging to subclasses IV and V. The members of subclass I lacked motifs 3, 5, and 7, while motif 8 was specific to subclass V (Figure 3c and Figure 4). The distributions of conserved motifs within the same branch were similar, and this supports the classification of the evolutionary tree.

### 3.5. Synteny Analysis for TCP Genes in Rose and Other Species

**Figure 5:**
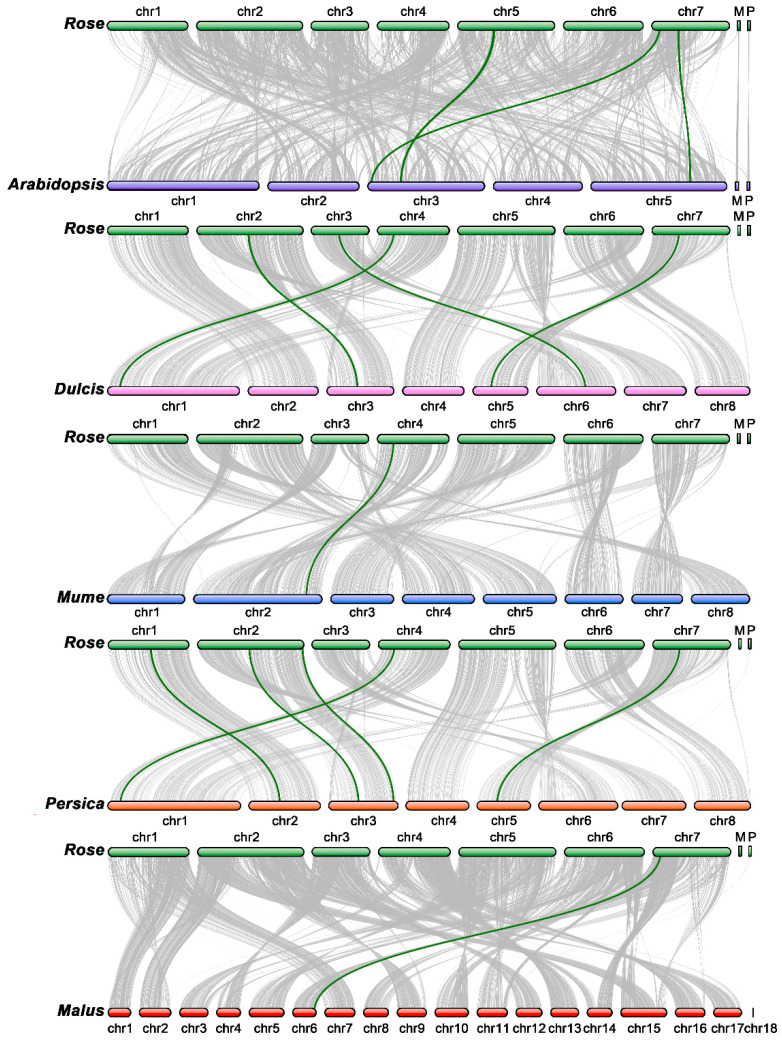
Synteny analysis of the TCP family. Green lines are the syntenic TCP gene pairs for different species. Gray lines are collinear blocks for plant genomes. The species names are *Rosa chinensis*, *Arabidopsis thaliana*, *Prunus dulcis*, *Prunus mume*, *Prunus persica*, and *Malus domestica Golden*, with the respective prefixes ‘Rose’, ‘Arabidopsis’, ‘Dulcis’, ‘Mume’, ‘Persica’, and ‘Malus’.

To study the collinearity relationships between the rose TCP genes, we used *Arabidopsis thaliana* and five species belonging to the Rosaceae family, including *Malus domestica Golden*, *Prunus mume*, *Prunus persica*, *Prunus dulcis*, and *Prunus avium*. These five species have a common ancestor. Additionally, *Arabidopsis thaliana* was used as a model organism for comparative genomics analysis. Fourteen collinear gene pairs were identified among these six species (Figure 5), with three homologous gene pairs between rose and Arabidopsis: PRQ31080/AT3G15060.1, PRQ16347/AT3G02150.2, and PRQ17858/AT5G52020.1. One homologous gene pair was identified between rose and malus (PRQ31080/mRNA:MD03G0194900), two were identified between rose and mume (PRQ17858/XP_016651394.1 and PRQ37082/XP_008223917.1), five were identified between rose and peaches (PRQ57759/ONI22810, PRQ50062/ONI16854, PRQ54300/ONI19971, PRQ37082/ONI27155, and PRQ17858/ONI06903) and four were identified between rose and dulcis (PRQ50062/XP_034208722.1, PRQ43584/XP_034218767.1, PRQ17858/XP_034214554.1, and PRQ37082/XP_034200985.1).

### 3.6. Analyses of Gene Expression of TCPs in Roses

**Figure 6:**
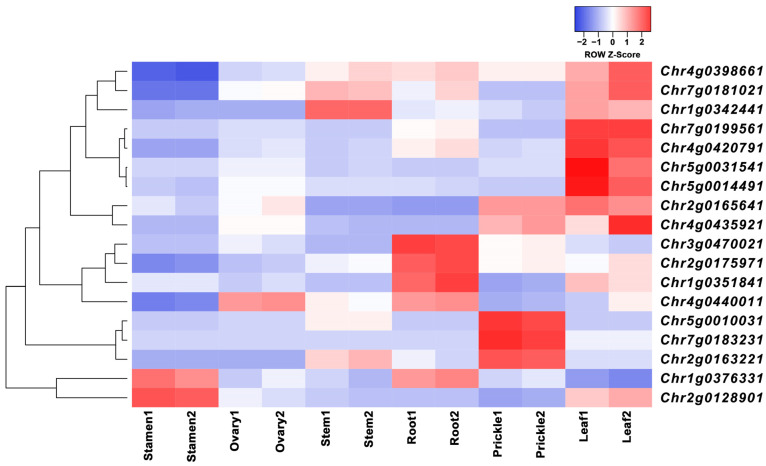
Heat map of TCP gene family members expression data in different rose tissues.

Expression data for the eighteen TCP genes were obtained from the rose transcriptome. The expression clustering analysis of TCP gene members was performed on the leaves, roots, prickles, stems, ovaries, and stamen tissues from *R. chinensis* “Old Blush”. Figure 6 shows the expression levels of the 18 genes in different tissues, with similar colors indicating similar expression patterns. TCP gene members are mainly expressed in the leaves, prickles, and roots. In the leaves, *Chr4g0398661*, *Chr7g0181021*, *Chr1g0342441*, *Chr7g0199561*, *Chr4g0420791*, *Chr5g0031541*, *Chr5g0014491*, *Chr2g0165641*, and *Chr4g0435921* exhibit the highest expression levels. In the roots, *Chr3g0470021*, *Chr2g0175971*, *Chr1g0351841*, and *Chr4g0440011* are highly expressed, while *Chr5g0010031*, *Chr7g083231*, and *Chr2g0163221* exhibit the highest expression levels in the prickles, indicating that each tissue has its own specific set of highly expressed TCP genes. In addition, the TCP gene expression level is relatively low in the stems, ovaries, and stamen tissues. In the stamens, only *Chr1g0376331* and *Chr2g0128901* have high expression levels, and these two genes are also highly expressed in roots and leaves. *Chr4g0440011*, is highly expressed in the ovaries, while *Chr7g0181021*, and *Chr1g0342441* are expressed in the stems. Some genes are almost not expressed in the ovaries, such as *Chr7g0181021*, *Chr5g0014491*, and *Chr4g0435921*, while these three genes are highly expressed in the leaves. *Chr5g0031541* and *Chr5g0014491* are only expressed in the leaves, belonging to specific highly expressed TCP genes in the leaves.

### 3.7. Expression Characteristics of Rose TCP Genes

**Figure 7:**
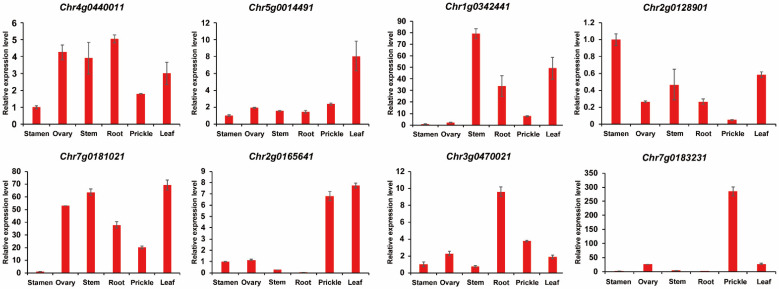
The expression of eight genes in rose organs including stamens, ovaries, stems, roots, prickles, and leaves.

We selected eight different TCP genes and analyzed their expression in different rose tissues (Figure 7). According to the results of transcriptome analysis, three of the eight representative genes are expressed in specific tissues, and the remaining five genes are expressed in multiple tissues. In the roses, the expression levels of TCP family genes in multiple tissues are consistent with the results of the transcriptome analysis. The genes that are almost not expressed in the stamens include *Chr7g0181021*, *Chr1g0342441*, and *Chr7g0183231*, and the gene with the highest expression level is *Chr2g0128901*, while *Chr5g0014491*, *Chr2g0165641*, *Chr3g0470021*, and *Chr4g0440011* are expressed at an intermediate level in the stamen tissue. All the genes are highly expressed in leaves, but *Chr3g0470021* and *Chr7g0183231* are expressed at an intermediate level. In the roots, five genes are highly expressed, including *Chr7g0181021*, *Chr1g0342441*, *Chr3g0470021*, *Chr4g0440011*, and *Chr2g0128901*, as well as *Chr5g0014491*, which is expressed at an intermediate level, while *Chr2g0165641* and *Chr7g0183231* are almost not expressed. In the ovary tissue, half of the genes are highly expressed, including *Chr7g0181021*, *Chr3g0470021*, *Chr4g0440011*, and *Chr2g0128901*. *Chr5g0014491* is expressed at an intermediate level, while many genes are expressed at a low level, including *Chr2g0165641*, *Chr3g0470021*, and *Chr7g0183231*, and the rest are highly expressed in the stem tissues. In the flower tissue, *Chr1g0342441* and *Chr2g0128901* are expressed at an intermediate level, while the rest are expressed at a relatively high level.

## 4. Discussion

The TCP gene family is relatively large and diverse, with many members having been identified across various plant species. Specifically, different members of the TCP gene family regulate various aspects of plant development. For leaf development, TCP genes control leaf morphology, size, and shape [43]. They are usually involved in regulating floral organ identity, size, and symmetry and promote flower development [16]. Some TCP genes play a critical role in hormone signaling pathways and responses to environmental cues [44]. Overall, the TCP gene family is essential for proper plant growth, development, and adaptation to the environment. 

The TCP gene family is characterized by the presence of a conserved DNA-binding domain called the TCP domain. The CDS structure of the TCP gene family is relatively simple, with few introns and exons. Evolutionary analyses showed that proteins on the same branch have similar CDS structures. Motif sequence analyses revealed that logo1 and logo2 are present in all the TCP proteins, indicating that the structures of these two motifs may be important for TCP protein functions. Based on the evolutionary classification, proteins on the same branch have very similar motif structures. For example, two proteins in class I contain TCP-specific logo3, which is not present in the other TCP proteins. The subcellular location of TCP proteins can vary depending on the specific protein and the context of its expression. However, some studies have indicated that many TCP proteins are localized in the nucleus, where they function as transcription factors to regulate gene expression. Specifically, TCP proteins contain a conserved DNA-binding domain, known as the TCP domain, which facilitates their binding to DNA target sequences in the nucleus [45]. In our research, the prediction of the subcellular localization of TCP proteins showed that all the TCP proteins are localized in the nucleus.

The TCP gene family is believed to have evolved due to a combination of gene duplication events and sequence diversification over time [46]. The TCP domain, which is a conserved DNA-binding motif found in all TCP proteins, is thought to have originated from a single ancestral gene and then been duplicated multiple times throughout its evolution [35,47,48]. These gene duplications are believed to have occurred at various points in its evolutionary history, resulting in the expansion of the TCP gene family across different plant species [25]. Despite the overall conservation of the TCP domain, individual members of the TCP gene family can vary greatly in their sequences and functions. This variation is thought to have arisen due to both random mutations and selective pressures, which acted on the different TCP genes to produce diverse protein structures and regulatory functions [25]. As a result, some TCP genes are highly conserved across different species, while others have diverged significantly and acquired new roles in plant development and physiology. Collinearity refers to the positional relationship between genes on the same chromosome, which is caused by the conservation of genes and the relative order among different species that originated from the same ancestral type [34]. The collinearity analysis of the TCP gene family in roses and five other species shows that there are relatively fewer collinear genes compared to those of other species, indicating that the TCP gene family has accumulated more mutations during evolution, resulting in fewer conserved fragments with shared features. As the species evolved, the gene differences within the TCP gene family became greater, which may have led to the emergence of new gene functions.

A previous study reported that class II TCP genes for ATTCP2, 3, 4, 10, and 24 in Arabidopsis encode transcription factors that coordinate growth processes during leaf development [49]. In our study, these class II TCP genes in Arabidopsis and PRO16347 (*Chr7g0183231*), PRQ53385 (*Chr2g0165641*), PRQ29494 (*Chr5g0014491*), PRQ29084 (*Chr5g0010031*), PRQ39050 (*Chr4g0420791*), PRQ31080 (*Chr5g0031541*), and PRQ50062 (*Chr2g0128901*) in rose belong to class IV, and the expression results indicate that PRQ53385 (*Chr2g0165641*), PRQ29494 (*Chr5g0014491*), PRQ39050 (*Chr4g0420791*), PRQ31080 (*Chr5g0031541*), and PRQ50062 (*Chr2g0128901*) have a high expression level. Therefore, we suggest that class V genes are likely to play a role in leaf development in roses.

The expression profiles of eight TCP gene family members in different rose tissues, including the roots, stems, leaves, ovaries, prickles, and stamens, were detected using qPCR. *Chr7g0181021* was almost not expressed in the stamens and was expressed at the highest level in the leaves, which is consistent with the results of transcriptome and fluorescence quantification. The fluorescence quantification and transcriptome analysis results showed that *Chr1g0342441* is expressed at the highest level in the stems and leaves and is almost not expressed in the stamens and carpels, indicating that this gene is mainly expressed in these two tissues. The qPCR results showed that *Chr2g0165641* is mainly expressed in the flower and leaf tissues and expressed the least in roots, which is highly consistent with transcriptome analysis results. *Chr5g0014491* is mainly expressed in the leaves, according to the qPCR results, while other tissues are poorly expressed. The qPCR results for *Chr3g0470021* showed that it is mainly expressed in the root tissue, while *Chr4g0440011* is mainly expressed in the ovaries and root tissues. The qPCR results for *Chr7g0183231* showed that it is mainly expressed in prickle tissue, while *Chr2g0128901* is mainly expressed in the stamen and leaf tissues. These results indicate that the qPCR results are consistent with the transcriptome analysis, and different genes have different expression levels, possibly due to functional differences.

In this study, we subsequently selected eight genes for qPCR, of which three belong to class IV: *Chr7g0181021*, *Chr3g0470021*, and *Chr4g0440011*. Four genes belong to class V: *Chr5g0014491*, *Chr7g0183231*, *Chr2g0128901*, and *Chr2g0165641*. *Chr1g0342441* belongs to class II. There are no genes belonging to class II in Arabidopsis thaliana, suggesting the possibility of functional diversity or gene loss during the process of evolutionary replication, which requires further validation. In class IV, all genes are expressed in the roots, and there are also gene expressions in the leaves, stems, and ovaries. *Chr7g0181021* is in the same branch as *ATTCP14* (AT3G47620.1). *ATTCP14* is a transcription factor that regulates seed germination [50,51] and stamen filament elongation and affects gene expression in radicles in a non-cell-autonomous manner [52]. *ATTCP14* is predominantly expressed in the vascular tissue of the embryo and affects gene expression in radicles [53]. In class V, all four genes are expressed in leaves and flowers. They are in the same branch as the Arabidopsis thaliana genes *ATTCP4* (AT3G15030.1), *ATTCP5* (AT5G60970.1), *ATTCP39* (AT5G08070.2), *ATTCP17* (AT5G08070.1), and *ATTCP13* (AT3G02150.1), which were involved in heterochronic control of leaf differentiation. TCP family proteins are involved in the heterochronic regulation of leaf differentiation [54,55,56,57]. *ATTCP4*, together with *ATTCP3*, *ATTCP24*, enhances hypocotyl cell elongation by directly activating YUCCA5 [58].

According to the analysis of the TCP gene family evolution, the TCP family is widely distributed in different species. The gene family members show differential expression levels and functional diversity in different plant tissues. The TCP gene family plays an important role in biological evolution and adaptation, and the functional diversity and expression pattern changes of family members reflect the adaptive capacity of organisms to environmental changes during evolution. In conclusion, gene family evolution analysis is of significant importance in elucidating biological evolution, predicting gene functions, and comparing interspecies differences.

## 5. Conclusions

Taken together, the main conclusion of our study is that the diversity of TCP gene function may be due to gene duplication or loss during the evolutionary process. The members of the TCP family are widely distributed in rose genomes, and genes within the same evolutionary classes share similarities in their structures. TCP gene family members are expressed in different tissues and may participate in various growth and development processes, such as root development, leaf morphology, and flower development. However, further supplementary research is necessary to explore this possibility. Currently, there is limited research on the TCP gene family in roses. Research of TCP gene family members provides a theoretical basis for breeding high-quality rose ornamental and application varieties in the future.

## Figures and Tables

**Table 1:** Physiological and biochemical characteristics of TCP gene family members.

Protein ID	Gene ID	Length of CDS (bp)	Number of Amino Acids	Molecular Weight (Da)	Theoretical pI	Predicted Subcellular Localization
PRQ40804	*Chr4g0440011*	1278	425	44,862.52	6.57	Nucleus
PRQ16145	*Chr7g0181021*	1281	426	45,529.8	6.81	Nucleus
PRQ31080	*Chr5g0031541*	1317	438	48,250.47	6.68	Nucleus
PRQ29494	*Chr5g0014491*	1326	441	47,923.63	8.42	Nucleus
PRQ40431	*Chr4g0435921*	1347	448	50,039.06	9.34	Nucleus
PRQ39050	*Chr4g0420791*	873	290	32,732.35	9.08	Nucleus
PRQ54300	*Chr2g0175971*	1008	335	35,745.35	9.02	Nucleus
PRQ56906	*Chr1g0342441*	1083	360	39,668.36	8.4	Nucleus
PRQ53141	*Chr2g0163221*	942	313	35,026.29	9.26	Nucleus
PRQ50062	*Chr2g0128901*	1095	364	39,858.69	6.45	Nucleus
PRQ59998	*Chr1g0376331*	981	326	33,999.1	6.32	Nucleus
PRQ57759	*Chr1g0351841*	924	307	32,831.23	8.57	Nucleus
PRQ17858	*Chr7g0199561*	1182	393	40,894.44	6.4	Nucleus
PRQ43584	*Chr3g0470021*	750	249	26,700.61	7.29	Nucleus
PRQ37082	*Chr4g0398661*	831	276	29,026.25	9.2	Nucleus
PRQ29084	*Chr5g0010031*	741	246	28,087.84	5.09	Nucleus
PRQ16347	*Chr7g0183231*	1155	384	42,654.47	8.93	Nucleus
PRQ53358	*Chr2g0165641*	1104	367	40,483.52	6.48	Nucleus

## Supplementary Materials

The following supporting information can be downloaded at: https://www.mdpi.com/article/10.3390/cimb45080401/s1, Table S1: TCP genes in the five species; Table S2: Primer sequences used in the qPCR.
